# Circulating Mitochondrial DNA Stimulates Innate Immune Signaling Pathways to Mediate Acute Kidney Injury

**DOI:** 10.3389/fimmu.2021.680648

**Published:** 2021-06-24

**Authors:** Jiaye Liu, Zhanjun Jia, Wei Gong

**Affiliations:** ^1^ Nanjing Key Lab of Pediatrics, Children’s Hospital of Nanjing Medical University, Nanjing, China; ^2^ Jiangsu Key Laboratory of Pediatrics, Nanjing Medical University, Nanjing, China; ^3^ Department of Nephrology, State Key Laboratory of Reproductive Medicine, Children’s Hospital of Nanjing Medical University, Nanjing, China

**Keywords:** acute kidney injury, mitochondrial DNA, STING, TLR9, NLRP3

## Abstract

Mitochondrial dysfunction is increasingly considered as a critical contributor to the occurrence and progression of acute kidney injury (AKI). However, the mechanisms by which damaged mitochondria mediate AKI progression are multifactorial and complicated. Mitochondrial DNA (mtDNA) released from damaged mitochondria could serve as a danger-associated molecular pattern (DAMP) and activate the innate immune system through STING, TLR9, NLRP3, and some other adaptors, and further mediate tubular cell inflammation and apoptosis. Accumulating evidence has demonstrated the important role of circulating mtDNA and its related pathways in the progression of AKI, and regulating the proteins involved in these pathways may be an effective strategy to reduce renal tubular injury and alleviate AKI. Here, we aim to provide a comprehensive overview of recent studies on mtDNA-mediated renal pathological events to provide new insights in the setting of AKI.

## Introduction

Acute kidney injury (AKI) is a cluster of clinical syndromes characterized by a rapid decline in renal function over short period of time, such as hours or days (1). The incidence of AKI is increasing with the aging population and leads to high mortality and disability, especially in patients in intensive care units (ICUs) (2, 3). Unfortunately, AKI is now still monitored by urine volume and serum creatinine level; indexes for early detection remain to be discovered. Additionally, there is a lack of effective therapeutic strategies for AKI (4). Therefore, studies on new diagnostic markers and treatment approaches are urgently needed (5).

Mitochondria are well known as energy-producing organelles. In addition to their canonical function to meet the energy requirements of cells, mitochondria also control the innate immune responses to sterile and infectious insults (6). In particular, mitochondria are essential for maintaining renal tubular cell survival and normal function but are highly susceptible to damage (7). Mitochondrial dysfunction is a crucial pathogenic factor which leads to tubular injury (8). The multiple pathological features related to tubular cell injury, such as oxidative stress, immune cell recruitment, inflammatory cytokine accumulation, and apoptosis, could all be caused by mitochondrial dysfunction (9). Pathological analysis of renal biopsy samples obtained from septic patients in the ICU supported the view that mitochondrial injury contributes to the pathogenesis of sepsis-causing AKI (10). Recent evidence suggests that mitochondrial damage occurs at early stage of AKI and even before the tubular cell apoptosis is detectable (11–13). Strategies for mitochondrial protection could effectively protect against AKI (14, 15). However, the mechanisms by which mitochondrial dysfunction promotes tubular cell injury and AKI progression are complex and need further study (16).

### Circulating mtDNA Serves as a Universal Danger-Associated Molecular Pattern in AKI

In addition to nuclei, mitochondria are the only organelles that contain DNA in eukaryotic cells. Mitochondrial DNA (mtDNA) is a circular double-stranded DNA (dsDNA) that encodes enzyme proteins, related ribosomal RNA, and transfer RNA required for various steps of oxidative phosphorylation (17). Normally, mtDNA is present in the mitochondrial matrix, but in cases of membrane potential reduction and/or mitochondrial membrane integrity damage, mtDNA can be translocated from the mitochondria to the cytoplasm (18). Free in the cytoplasm, mtDNA can serve as a danger-associated molecular pattern (DAMP) to trigger the innate immune system to initiate a non-infection-related inflammatory response, and that could mediate local inflammation, cell death, tissue injury, and dysfunction (6, 19). Moreover, biological behaviors, such as cell necrosis and pyroptosis that damage the structure of the cell membrane, can cause mtDNA to be released from the intracellular and enter the extracellular environment, such as blood or urine, which results in systemic inflammation and injury (20). As a newly identified mitochondrial DAMP, mtDNA has attracted growing interest in clinical and basic research, including studies on AKI in recent years.

In early AKI, pathological changes in the mitochondria are found in multiple kinds of AKI, including the septic, ischemic and toxic insults in origin (8, 21, 22). Circulating mtDNA in AKI patients’ blood or urine has been investigated in numerous studies. As reported, the plasma mtDNA quantity was enhanced in circulation after trauma and was associated with the mortality of patients in ICUs (23, 24). MtDNA in plasma has been suggested to be a promising biomarker in the clinical context of AKI and severe lung injury (25, 26). Evidence from clinical samples showed that in addition to the circulation, mtDNA in urine is an important marker of renal injury and AKI progression (27–30). Urine mtDNA levels were significantly enhanced in patients with AKI and positively correlated with serum creatinine levels, urine neutrophil gelatinase associated lipocalin (NGAL), kidney injury molecule 1 (Kim1), and the levels of inflammatory factors (30). Animal experiments also confirmed that in the AKI mouse model, urine mtDNA was derived from mitochondria-damaged renal tubular cells and significantly and positively associated with serum creatinine and urea nitrogen levels (27). Moreover, *in vitro* experiments showed that mtDNA derived from necrotic cells induced inflammation in renal tubular epithelial cells (31–33). The above evidence suggests that free mtDNA is a significant damage factor during the occurrence and progression of AKI, and could serve as a novel predictor in AKI. Furthermore, strategies of targeting mtDNA-related pathways can be potentially applied in the treatment of AKI.

The innate immune system plays a crucial role in mediating tubular injury and renal dysfunction. During AKI, elevated cytokines produced through innate immune pathways are closely related to the renal pathological damage (34, 35). Moreover, mtDNA, as an identified DAMP, can trigger the activation of the innate immune system in numerous pathological conditions such as sepsis, AKI, liver failure, and lung injury (36–38). Considering the important contribution of mtDNA in AKI and its related innate immune pathways, we summarize the major signaling pathways which are triggered by mtDNA and the potential therapies based on these studies.

## Mechanisms Contributing to the mtDNA-Associated Pathogenesis of AKI

**Figure 1:**
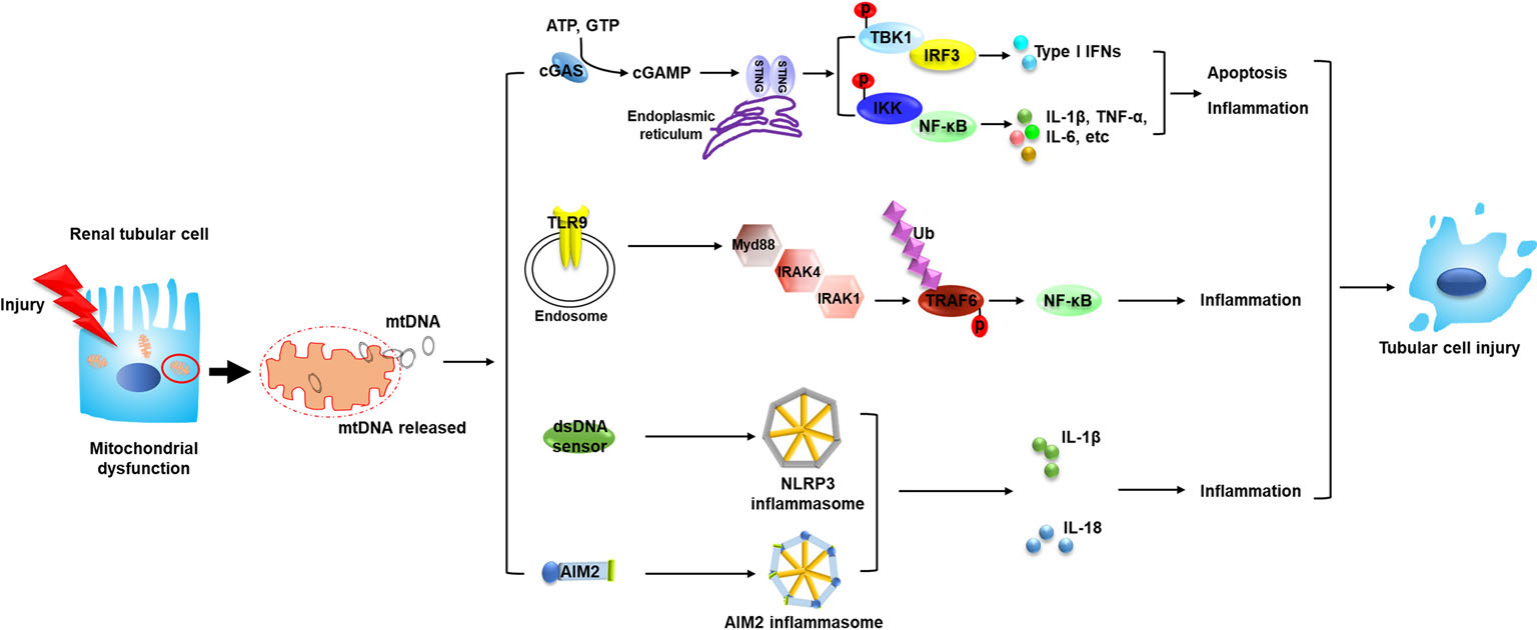
Circulating mtDNA triggers the innate immune system through several mechanisms and mediates the pathogenesis of AKI. The occurrence of AKI is closely associated with mitochondrial dysfunction in renal tubular cells. Tubular mitochondrial damage leads to mtDNA leakage into cytosol and extracellular space. Circulating mtDNA binds with different DNA sensors and activates several innate immune signaling pathways such as cGAS–STING, TLR9-Myd88-NF-*κ*B and NLRP3 inflammasome, leading to tubular inflammation and injury, which contributes to the progression of AKI. MtDNA, mitochondrial DNA; STING, stimulator of interferon genes; TBK1, TANK binding kinase 1; IKK, the I*κ*B kinase; p, phosphorylation; IRF3, interferon regulatory factor 3; NF-*κ*B, nuclear factor kappa B; TNF, tumor necrosis factor; TLR9, toll-like receptor 9; Myd88, myeloid differentiation factor 88; IRAK, interleukin 1 receptor associated kinase; Ub, ubiquitylation; TRAF6, TNF receptor associated factor 6; AIM2, absent in melanoma 2; IL, interleukin.

As a DAMP, free DNA is recognized and consequently elicits the related signals to transfer downstream through pattern recognition receptors (PRRs). MtDNA has been reported to be captured by different PRRs (6); various PRRs which could recognize mtDNA and their related pathways are reported to play vital roles in the progression of AKI, as discussed below (**Figure 1**).

### The cGAS–STING System

Stimulator of interferon genes (STING), also called mediator of IRF3 activation (MITA) or transmembrane protein 173 (TMEM173), was found to be a novel DNA recognition receptor. Since STING was discovered in 2012, an increasing number of studies have confirmed the existence of the cGAS–cGAMP–STING pathway in different conditions and its vital role in the removal of DNA viruses (39, 40).

The cGAS–STING system starts with the recognition of DNA. DNA is bound to cyclic GMP-AMP synthase (cGAS) in a non-sequence-dependent way through its phosphate ribose skeleton to form a dimer, which can catalyze the synthesis of 2′-3′ cyclic AMP-GMP (2′–3′ cGAMP), a secondary messenger in cells. Then, cGAMP is transferred to the joint protein STING and binds it through hydrophobic interaction and hydrogen (41, 42). Activated STING transfers to the Golgi apparatus, where two cysteine residues of STING (Cys88 and Cys91) undergo palmitoylation, which leads to the exposure of the active C-tail domain (CTD). The structure of CTD is similar to the substrate of TANK-bound kinase 1 (TBK1), and the combination with TBK1 activates the downstream pathways. Two main downstream pathways of STING have been recently described: 1) TBK1 mediates IRF3 phosphorylation, then initiates the transcription of type 1 interferon to produce IFN-*α* and IFN-*β*, and also induces many other target genes such as interleukin (IL)-6 and IL-12; 2) Stimulation of cGAS–STING activates the classical NF-*κ*B inflammatory responses, which is not fully dependent on the CTD of STING, to generate TNF-*α*, IL-Iβ, IL-6, and so on (43–48). Previous studies mainly focused on the antiviral effect of STING in removing DNA viruses. In fact, the cGAS–cGAMP–STING pathway plays many other roles in addition to its antiviral ability. For example, STING is important for many non-infection-related inflammatory states by mediating acute and chronic inflammatory injury and participating in the onset and progression of auto-inflammatory diseases (49). Strikingly, many studies have confirmed that not nuclear DNA but mtDNA is the key factor for triggering the cGAS–cGAMP–STING pathway, possibly because mtDNA has fewer DNA repair systems than nuclear DNA and is more prone to damage (50–54).

As in AKI, when the kidney suffers from cisplatin-induced injury, mitochondrial damage occurs accompanied by the increased mitochondrial membrane permeability, which causes the mtDNA to leak into the cytoplasm through the BAX pore on the outer mitochondrial membrane. After mtDNA leakage, the cGAS–STING pathway is activated, which leads to the phosphorylation of transcription factors, promotion of inflammatory factor secretion, and AKI progression. Activation of the cGAS–STING pathway has been observed in multiple AKI mice models and AKI patients (37, 55, 56). Moreover, STING knockout mice showed attenuated renal function, tubular injury, and inflammation when subjected to cisplatin treatment (37). In addition, STING also mediates the secondary renal inflammation and tubular injury (57). Although few studies focused specifically on the kidney injury in sepsis, a role for the cGAS–STING pathway in sepsis has been identified. Li N et al. found that STING can activate the expression of the NOD-like receptor family pyrin domain-containing-3 (NLRP3) inflammasome by promoting the nuclear translocation of phosphorylated IRF3, stimulating the expression of inflammatory factors in myocardial cells in LPS-induced septic mice and aggravating myocardial injury (58). Qiongyuan Hu et al. reported that STING signaling pathway in the intestinal tract was significantly activated, and the expression level of STING in the human intestinal lamina was related to intestinal inflammation in patients with sepsis and cecal ligation and puncture (CLP)-induced septic mice. Moreover, STING-knockout mice showed reduced bacterial translocation, decreased intestinal permeability, and a weaker inflammatory response, indicating that modulation of the mtDNA–STING pathway might facilitate healing of mucosa and defend the intestinal barrier in septic patients (59). However, STING-mediated specific downstream mechanism in AKI needs to be further explored.

As STING pathway is involved in the pathogenesis of AKI, STING inhibitors have been employed to explore their effects on AKI. To date, two kinds of STING antagonists have been tested in AKI mouse models and displayed beneficial effects towards ameliorating acute tubular injury and renal dysfunction (37, 60). Of note, there is a species difference between human STING and murine STING which is possibly due to the difference in the amino acid sequences between species and the different binding activities to specific cyclic dinucleotides (61, 62). Therefore, it is required to take this into account for the design of STING antagonists.

### TLR9 Signaling

Toll-like receptors (TLRs) are important pattern recognition receptors that regulate adaptive and innate immunity and mediate resistance to microbial invasion. To date, 12 TLRs have been found in mice, and 10 have been found in humans (63). TLRs can be categorized into cell-surface TLRs (TLR1, 2, 4, 5, and 6, which recognize fungal or bacterial products) and intracellular TLRs (TLR3, 7, 8, and 9, which recognize RNA and DNA products) (64). TLR9 is a cytoplasmic receptor for unmethylated CpG motif containing DNA (CpG-DNA) found in DNA viruses and microbial DNA (65). Generally, TLR9 is localized to intracellular membrane compartments, such as the endoplasmic reticulum, endosome, and lysosome. Therefore, clathrin-mediated endocytosis is needed for the DNA translocating from the cell surface to the intracellular compartment and binding with TLR9 (66–68). Recent studies suggest that mtDNA can be recognized by TLR9, which triggers MYD88-dependent NF-*κ*B-mediated gene transcription and leads to inflammation and apoptosis in acute lung injury, non-alcoholic steatohepatitis, and heart injury (26, 69, 70). Besides, mtDNA is also recognized following endocytosis and binding to TLR9 (71, 72).

In AKI, *in vivo* studies have illustrated that TLR9 contributes to the development of septic and ischemic renal tubular injury by promoting inflammation, apoptosis, and necrosis (73–76). Meanwhile, enhanced levels of mtDNA were found in the peritoneal cavity and plasma of septic mice induced by CLP or LPS. Furthermore, renal function injury, abnormal mitochondrial dysfunction and oxidative stress damage in the proximal tubules can be reversed by TLR9 knockout. More importantly, intravenous injection of mitochondrial debris (MTD) including substantial amounts of mtDNA in mice induced the similar inflammatory response to that of mice subjected to CLP; while knocking out TLR9 or DNase pretreatment attenuated this effect, which provided evidence that free mtDNA could mediate kidney injury through TLR9 (77). All these studies imply the role of mtDNA in the TLR9-mediated pathogenesis in AKI.

However, it is inconsistent whether the TLR9 pathway specifically causes tubular dysfunction in sepsis. Results from a previous study indicated that TLR9 was predominant on dendritic cells (DCs) in the interstitium but not on tubular epithelial cells (78). Consistent with this observation, Liu et al. documented that TLR9 expression was weakly detected in glomerular cells or renal tubular epithelial cells in control mice, although it was upregulated in tubular epithelial cells and glomerular cells in the mice subjected to CLP (79). Another study suggested that only the activation of TLR9 on renal tubular epithelial cells exacerbated the ischemic AKI; the activation of TLR9 on other cell types was renoprotective during AKI (80). These conflicting results suggest a necessity for further experimentation to clarify the mechanism of TLR9 activation in AKI. However, the endogenous mtDNA-mediated activation of TLR9 should contribute to the pathogenesis of AKI, and targeting TLR9 using siRNA or selective antagonist through targeting tubular delivery could protect against AKI (79, 81, 82).

### Activation of NLRP3 Inflammasomes

NLRP3 inflammasomes are mainly composed of the receptor protein NLRP3, adaptor protein ASC (apoptosis-associated speck-like protein containing a CARD) and downstream caspase-1 (83). Studies have verified the vital roles of activated inflammasomes in numerous pathological conditions, such as metabolic, autoimmune, autoinflammatory and infectious diseases (84–88). NLRP3 inflammasomes are mainly expressed in monocytes, macrophages, neutrophils, dendritic cells, and many non-hematopoietic cells (89, 90). Once activated, NLRP3 can trigger the self-cleavage and maturation of caspase-1, then activated caspase-1 not only promotes the maturation and secretion of various pro-inflammatory cytokines, including IL-1β and IL-18, but also triggers pyroptosis, which can eliminate pathogens and damaged cells (91, 92). Notably, NLRP3 inflammasomes are able to recognize various patterns that are closely related to many diseases, such as bacterial polypeptide antibiotics, bacterial RNA, and influenza virus ion channel proteins, ATP, and mtDNA (93–99). Although NLRP3 inflammasome activation triggered by cytosolic mtDNA has been demonstrated in many cases (98, 100, 101), direct evidence of mtDNA triggering NLRP3 inflammasomes in AKI is lacking. However, studies found that patients in the ICU had high levels of free mtDNA and IL-18 in the plasma, which were positively associated with the severity and mortality of diseases (25, 102), which suggested that cytosolic mtDNA might drive inflammasome activation and IL-18 secretion in an indirect or possibly a direct manner.

### Others

In addition to the aforementioned sensors and adaptors, studies have found other kinds of DNA sensors. However, not all receptors can combine with mtDNA directly. Absent in melanoma 2 (AIM2), a member of the ALR family, is composed of the DNA-binding HIN domain and pyrin-signaling domain (PYD). Cytoplasmic dsDNA can bind to the HIN domain of AIM2 non-specifically, which stimulates the assembly of AIM2 in inflammasomes. Activation of the AIM2 inflammasome promotes apoptosis and the maturation of pro-inflammatory cytokines IL-18 and IL-1β (103, 104). However, the minimum number of base pairs of DNA that can be recognized by AIM2 is 80 kb (105), while mtDNA is a short strand of nucleic acid, at 17 kb (47), which cannot effectively bind to AIM2. AIM2 has a protective effect against microbial infection, but it is pathogenic to sterile inflammatory diseases, such as cardiovascular diseases, skin diseases, neuroinflammatory diseases, and CKD. During the pathogenesis of CKD, DNA from necrotic cells in the kidney was recognized by AIM2, which induced the assembly of the inflammasome complex and activated macrophages to produce IL-18 and IL-1β (106). Therefore, we hypothesize that AIM2, although unable to bind with mtDNA, may mediate the similar inflammatory effect in renal injury by responding to nuclear DNA. In addition, there are some other sensors that might recognize cytosolic mtDNA, such as DNA-dependent activator of IFN-regulatory factors (DAI) and RNA polymerase III. However, further studies are needed to explore their binding with mtDNA and/or the function in the pathogenesis of AKI (107, 108).

## Summary and Future Perspectives

Mitochondrial injury is a common pathological phenomenon in different types of AKI models, which leads to the release of mtDNA into the cytoplasm. Currently, circulating mtDNA from damaged mitochondria has been reported to be involved in the pathogenesis of AKI. Clinical studies demonstrated that level of free mtDNA was elevated in AKI patients and was associated with the disease severity and prognosis (28, 29), suggesting that circulating mtDNA has the potential to serve as a predictor for AKI. As one kind of mitochondrial DAMPs, mtDNA could promote inflammatory response through binding with the DNA sensors and triggering the innate immune activation. Although the identified sensors of mtDNA could also recognize other kinds of nucleic acids, studies have mentioned the importance of mtDNA, but not nuclear DNA, in the occurrence and the development of AKI (38). Furthermore, depletion of mtDNA markedly attenuated the acute renal tubular cell injury (37), indicating that strategies targeting both mtDNA-mediated pathways and mtDNA clearance mechanisms need to be considered in AKI therapy. However, the detailed mechanism underlying mtDNA-mediated pathogenesis in AKI and the strategies antagonizing mtDNA-associated kidney injury need to be further explored.

## Author Contributions

WG and ZJ conducted a review of articles. JL and WG wrote the manuscript. All authors contributed to the article and approved the submitted version.

## Funding

This work was supported by grants from the National Natural Science Foundation of China (81873599, 81970581 and 82070701), and the Nanjing Medical Science and Technique Development Foundation (QRX17166).

## Conflict of Interest

The authors declare that the research was conducted in the absence of any commercial or financial relationships that could be construed as a potential conflict of interest.
